# The interaction between actin and FA fragment of diphtheria toxin

**DOI:** 10.1007/s11033-012-2387-0

**Published:** 2012-12-28

**Authors:** A. Ünlü, M. Bektaş, S. Şener, R. Nurten

**Affiliations:** 1 Medical Faculty Department of Biophysics, Trakya University, Edirne, Turkey; 2 Istanbul Faculty of Medicine, Department of Biophysics, Istanbul University, Istanbul, Turkey

**Keywords:** Actin, Diphteria toxin, Protein, Protein interactions, Three dimensional structure of proteins

## Abstract

Actin protein has many other cellular functions such as movement, chemotaxis, secretion and cytodiaresis. Besides, it have structural function. Actin is a motor protein that it has an important role in the movement process of toxin in the cell. It is known that F-actin gives carriage support during the endosomal process. Actin is found in globular (G) and filamentous (F) structure in the cell. The helix of actin occurs as a result of polymerisation of monomeric G-actin molecules through sequential rowing, is called F-actin (FA). Actin interacts with a great number of cellular proteins along with cell skeleton and plasma membrane. It is also known that some bacterial toxins have ADP-ribosylation affect on actin. Diphteria toxin is the part which has the FA enzymatic activity corresponding the N-terminal section of the toxin, which inhibits the protein synthesis by ADP-ribosylating the elongation factor 2 in the presence of NAD. FA, taken into the cell by endocytosis inhibits protein synthesis by ADP-ribosyltransferase activity and breaks the cytoskeleton. In the studies both in vitro and in vivo, actin with interaction FA of diphteria toxin has been yet to be fully elucidated. The aim of this study was to determine the three dimensional structures of actin with interaction FA of diphteria toxin by the amprical methods and in paralel with the computing technology, theoretical methods have gained significant importance. In our study, actin with interaction FA of diphteria toxin has been determined as the most possible interaction area with the theoretical method; analogy modelling. This area has been closed in the presence of polypeptides and FA-actin interactions have been tested with the gel filtration chromatography techniques. As a result of the findings, we found that 15 amino acid artificial peptides (DAMYETMAQACAGNR) corresponding to 201–215 amino acid residues of FA interacts with G-actin and closes this area. Secondly, in the model formed with the analogy modelling, it appears that the most possible interaction area is between FA (tyr204) and G-actin (gly48). Results obtained from both theoretical and experimental data support the idea that the interaction occurs in this area.

## Introduction

Proteins have important functions in the cells. Their settlement, patterns, expressions, structures, interactions and folding patterns in the cells are vitally important. Although the arrays making protein up provide important information, high variability of them makes it impossible to gain enough functional information. For this purpose, it has been tried that three dimensional structures of proteins are determined. Folding patterns of proteins according to changing ambient conditions enables their interactions with each other. In order to determine protein–protein interaction, the simulation of these proteins must be designed. By reflecting cause and effect relation, which belongs to theoretic and physical systems, to a computer model, simulation is a kind of modelling technique which enables real system behaviours to be seen on computer model under changing conditions. The solution of three dimensional structures of proteins is achieved by X-ray crystallography and nuclear magnetic resonance (NMR) methods. The most reliable way to form three dimensional structure of protein, whose structure is unknown, is homology modelling. It is also known as comparative modelling. Using comparative modelling has led to significant increases in the number of three-dimensional models of proteins. Eukaryotic cells have advanced internal supporting structures which are also known as cytoskeleton. Basically, cytoskeleton structures consist of microtubules, inter-mediate filament and actin filament. Actin filament is found in shapes of globular (G) and filamentous (F) in the cell. Actin protein has many other cellular functions such as movement, chemotaxis, secretion and cytodiaresis. Besides, it have structural function. Actin is a motor protein that it has an important role in the movement process of toxin in the cell. The active form of actin is a helical polymer called F-actin, assembled from monomeric subunits of G-actin. Actin creates 5 % of all proteins in eukaryotic cells, and about 20 or 25 % of muscle proteins [1]. G-actin is a global protein weighting 42.500 Da. The primary structure of it contains 375 amino acids, and there are great similarity between species (homology). The primary structure of actin consists of four subunits [2]. The primary subunit includes sequences of 1–32, 70–144, 338–375 amino acids, the second subunit includes sequence of 33–69 amino acids, the third subunit includes sequences of 145–180, 270–337 and the fourth subunit includes sequence of 181–269 amino acids. Each G-actin molecule can bind to an ATP molecule. G-actin molecule may undergo post-synthesis modifications such as acidification from N-terminus and ADP-ribosetion [3]. G-actin molecules can bind to ATP, Ca and Mg [4]. In physiological ionic conditions and in presence of magnesium and ATP, G-actin molecules polymerized ‘un-covalently’ in order to make a couple of helix filaments for F-actin. About 50 % of actin molecules in animal cells are monomer-structure. G-actin is form of free monomer or small complexes with certain proteins. There is a dynamic equilibrium between G-actin and F-actin molecules. This helps many cellular functions including cell-surface movement to happen [5, 6]. Actin interacts with many cellular proteins. Besides, it is cytoskeleton and plasma membrane. This proteins include in Eukaryotic elongation factor 1 (eEF1) and eukaryotic elongation factor 2 (eEF2), Deoxyribonuclease I (DNase I) responsible for synthesis of proteins [7, 8]. In addition, actins of certain bacterial toxins have been reported that they make actin change into ‘ADP-ribose’ [9]. It was shown that DT catalyzes transfer of ADP-ribose moiety of NAD into a covalent linkage with eukaryotic elongation factor 2 (eEF2). ADP-ribosylation results in inactivation of eEF2 and inhibition of protein synthesis, respectively. ADP-ribosyltransferase activity resides in the N-terminal FA (21 kDa) [10–12]. Fragment B is involved in receptor binding on the cell surface. Following receptor binding, the holotoxin is cleaved into the two fragments with transfer of FA into the cytosol via an endocytic process, inhibits synthesis of protein by activity of ADP-ribose transferase. The inhibition of protein synthesis due to ADP-ribosylation of eEF2 has been regarded as the primary event in DT induced cytotoxicity. Generated from DT effect, DNA fragmentation and cytoskeleton destruction have been reported [13]. FA fragment of diphtheria toxin destructs cytoskeleton by interacting with actin [14]. We aimed that Modelling of FA of diptheria toxin with interaction actin has been performed and also revealed interactions region from available models. In presence of synthetic polypeptides, FA-actin interactions has been tried to clarified by closing generated possible interaction areas after gel filtration chromatography techniques. FA recovery, in presence of trypsin of diphtheria toxin, carried out via methods of post-ingestive electrophorese or gel filtration. By interacting G-actin, in appropriate conditions, with polypeptides (six synthetic polypeptides which cover an area as large as around 100 amino acids cover) which have been synthesized abroad, areas on actin which FA ligated, have been closed down. At the end of study, synthetic polypeptide, removing FA-actin interaction, has been determined and interaction area on complex has been showed up.

## Materials and methods

### Purification of actin from rabbit striated muscle tissue

Globular actin monomer (G-actin) was prepared from rabbit skeletal muscle acetone powder by two polymerization/ultracentrifugation/depolymerization cycles [8]. It was further purified at 4 °C by gel filtration on Sephacryl HR S-100, equilibrated and run with 5 mM potassium phosphate, pH 7.5, 0.5 mM ATP, 0.1 mM CaCl_2_, 0.5 mM dithiothreitol, and 1 mM NaN_3_ and used either directly or after a short storage at 4 °C not exceeding a few days. G-actin was converted into the filamentous form (F-actin) by dialysis against polymerization buffer containing 10 mM NaCl, 3 mM MgCl_2_, 5 mM potassium phosphate, pH 7.5, 0.5 mM ATP, 0.1 mM CaCl_2_, 0.5 mM dithiothreitol, and 1 mM NaN_3_. The dialysis step was followed by incubation at room temperature for 30 min and then overnight at 4 °C. Solution was centrifuged in 80.000×*g* and 14 °C ultracentrifuge, and F-actins were precipitated. Pellet of F-actin was transferred into depolymerize buffer and lysed by homogenizer. Pellet of F-actin was centrifuged in 100.000×*g* for 45 min, supernatant liquid (S100) was passed through gel filtration colon (Sephacryl HR S-100), and G-actin was purified. Amount of purified G-actin was determined by spectroscopic methods.

### Exracting subunits of diptheria toxins via trypsin local digestion

Dipthera toxin was partly digested by trypsin to rate molar as 1/200 in presence of 50 mM Tris–HCl, pH 7.4, 250 mM sucrose, 7 mM MET, 0.2 mM PMSF. In order to stop the interaction, it was denaturazied with SDS after adding trypsin inhibitor originated from soy bean in rate of 1:1. FA fragment digested via trypsin was separeted by SDS PAGE electrophoresis and then FA fragment cut from gel and it was purified chromatographic methods. Samples were concentrated by vivaspin tubes (V-10.000) or liyophilizator. Amount of ADP-ribosylation and activity of FA, were tested by ELISA.

### Analysis of ADP-ribosylation

Diphtheria toxin-mediated ADP-ribosylation was carried out for 10 min at 20 °C in 25 μl reaction mixtures containing 50 mM Tris–HCl, pH 7.4, 7 mM mercapoethanol and, 5 mM [adenosine-^14^C] NAD (specific activity; 535 Cu/mol) and 120 μg/ml FA of diphtheria toxin. Following incubation, 5 μl aliquots were taken from the reaction mixtures and applied to GF/A (Whatman, Maidstone, UK) glass microfibre filters which were washed successively in cold 5 % TCA, ether-ethanol (v/v:1/1). After drying, filters were transferred to vials containing 5 ml 0.4 % 2,5-diphenyloxazol in toluene and TCA-precipitated radioactivity was determined in a liquid scintillation counter (Packard Tri-Carb 1000 TR) [11, 12].

### Gel filtration chromatography analysis

HiPrep Sephacryl S-100 (16 × 20 cm) colon and HiPrep Sephacryl S-200 (16 × 20 cm) colon were used in the study. It is determined that total volume of colons was (V_c_) 120 ml, and dead storage of them was (V_0_) 40 ml. Colons were calibrated with standarts of aprotinin (6,5 kDa), carbonic anhydrase (29 kDa), ovalbumin (43 kDa), BSA (66 kDa) and conalbumin (75 kDa); calibration graphic was designed by determining μ values which is gel area separation modulus according to arrival volume of standarts. μ values were measured by using [15] formula. After calibration process, FA and FB fragments of diptheria toxin were seperated and arrival volume of these fragments were checked on calibration graphic. In order to test the diptheria toxin’s reaction with actin, four peptides of dipheria toxin which were produced synthetically were passed through colon and arrival volume of them was measured. After samples were segmented as 0.5 ml at a pressure of 0.35 mPa, at a speed of 0.8 ml per min, optical density of samples was determined at 280 nm. At the end of study, colons were washed in 20 % ethyl alcohol solution. Radioactive labelling of FA was incubated in (50 mM Tris–HCl pH: 7.4); in presence of around 200 μM (FA), 3 ([^3^H]sodium borohidrit), and 20 °C. Some GF/C taken from dialysed sample after incubation was put into filter and radioactivity which was ligated to each protein, was measured as dpm. Labelled FA and G-actin interaction was examined in gel filtration chromatography (sephacryl S-100). After chromatography, radioactive content of samples was measured in 5 ml scintilation liquid.

### Liyophilisation of samples

Samples were covered by parafilm in ependorf or falcon tubes and stored at −80 °C. Before tubes were put into device, they were needled in order to provide them take air. Samples were liyophilised during the overnight by vaccummed under high pressure.

### Analysis of electrophorese

Samples were tested with sodium dodecyl sulphate poly acrylamide (SDS-PAGE) according to Laemmli method [16]. Silicone brackets were put between glass plates by adding 12 % volume seperation gel. In order to build a smooth gel surface, 1 centimeter water layer was built and waited to be polymerized. Water layer on gel was removed and 5 % volume batching gel was added. Pecten which would build the well where the samples would be put, were put between glass plates and waited to be polymerized. Samples which woul be given to electrophorese, were mixed with denaturing buffer at 1:1 rate and boiled for 2 min. Electrophorese chamber was filled with anode and cathode running buffers. During the installation process of sample, multi protein standart (Fermentas Protein Ladder), whose molecule weight was between 10 kDa and 260 kDa, was used in a well as molecular weight standarts. 80 voltage was applied for batching gel, and 100 voltage was applied for seperation gel. Voltage was stopped when labelling paint (Brom fenol blue) used during electrophorese process came to end of gel. Gel was taken from electrophorese device and put to be painted. Later, gel and, in the presence of 7 % acetic acid 30 % wool alcohol, extra paint were removed. Gel whose protein bands were painted, was put on Whatman filter paper, and covered with plastic wrap. Gel was dried by gel-drier at 80 °C for 1 h by being vaccumed.

### Immune-blotting method

After SDS-PAGE, unpainted gel was cut, and put into transferin buffer with nitrocellulose membrane and waited there for 30 s. After 3 MM Whatman filter paper was washed with transfering buffer, nitrocellulose membrane was put between 3 MM Whatman filter papers and transfering process was completed by dry-saturating device for 45 min at a pressure of 200 mA. After transfering process, in order to prevent unoriginal bondings, nitrocellulose membrane was vacillotary saturated with Tris buffered salt-solution (TBSS) including BSA at room temperature for 2 h. After membrane was washed three times with TBSS, it was diluted with 1 anticorp 1:500 TBSS-BSA and shaked for 2 h at room temperature. Membrane was washed three times with TBSS. After immune globuline G, second anticorps and united with alcaline phosphatase, was diluted with TBSS-BSA at rate of 1:1000 an put into medium, emmbrane was vacillotary waited at room temperature for 1 h. Again, membrane was washed three times with TBSS. Alcaline phosphatase substrate BCIP (150 μg/ml) and NBT (300 μg/ml) was resolved in substrate buffer and mixed with membrane. Mixture was shaked at night and building of bonding was provided. By adding stopping solution to membrane, reaction was stopped.

### Protein purification from SDS-PAGE

In the presence of trypsin, after partial digestion, FA fragment was given to SDS-PAGE gel. Gel was divided vertically into two parts. First part was painted as told in section analysis of electrophoresis and dried after the paint of it was removed; second part was sliced by 1 milimeter spaces and homogenized in (50 mM Tris–HCl, pH 7.5, 150 mM NaCl, 0.1 mM EDTA and 0.1 mg/ml BSA) at room temperature during the night. Silicon coated glass cotton and conical tubes were used and centrifugation lasted for 10 min at 3000×*g*, and then the liquid part was taken. Cold acetone (−20 °C) was put on it, and it was made precipitated. After 100 μl 6 M guanidin hydrocholeride was added on pellet, it was dialyzed during whole night against re-naturization solution (50 mM Tris–HCl pH 7.5, 150 mM KCl, 1 mM DTE, 0.1 mM EDTA, 0.1 mg/ml BSA, 20 % gliserol). After dialysis, by taken 10 μl sample, amount of FA was determined by ADP-ribosylation; and sample was concentrated by vivaspin tubes (V-10.000).

### Interaction of G-actin and FA

After amounts of purified FA and G-actin were determined by spectroscopic and ELISA methods, interaction started and lasted for 1 or 2 h at a rate of 1:1 in medium of G-actin at 20 °C. Similarly, [^3^H]FA was also involved in interaction with G-actin and resolved at gel filtration chromatography. By determining optical density and radioactivity of the samples, the interaction of G-actin and FA was shown.

### Theoretical calculation of protein–protein interactions

X-ray crystallography or PDB folders formed by NMR of primary sequences (patterns) of proteins whose interaction would be determines, were found on www.expasy.org (Expert Protein Analysis System) and www.pdb.org (Protein Data Bank). Academic version of protein analysis softwares such as Pymol, Rasmol were used, and possible interaction surfaces were displayed by mapping related residues of proteins. Proteins, whose patterns were determined before, was put into interaction at ClusPro 2.0 simulation software is readily available on protein–protein docking system at Structural Bioinformatics Laboratories of Boston University. This software is a protein docking software which is Fast Fourier Transform (FFT) correlation approach, and it has been expanded in order to use double-logical interaction potentials. The best 1000 energy conformations were clustered on this software to be used at possible interactions. First of all, for exploring interaction areas, energy area is widely researched by using a simplified energy model and the theory of restricted flexibility. After, determined areas were focused by using detailed scoring and sampling. Second step of algorithm is a step where clustering of structures for range measurement by using double logic RMSD (root mean square deviation). The biophysical meaning of clustering is to isolate energy basins of highly loaded energy areas. At this software, Fast Fourier Transform (FFT), which is a docking method with double logic potential appled against PIPER; DARS (Decoys as the Reference State), which is a method to produce reference contiditons for molecule identification potentials; a clustering technique for discovering of possible conformations; Semi-Definite programming based Underestimation (SDU) which provides energy optimization and removing of nonlocal clusters by analyzing free-energy stability are respectively used [17]. By evaluating ten interaction areas according to termodynamical energy calculations, areas where possibility of bonding is high, were determined.

## Results

### Determination of structures of diptheria toxin and G-actin by theoretical method

#### Modelling of subunits of diptheria toxin by using the primary sequences of It

**Fig. 1:**
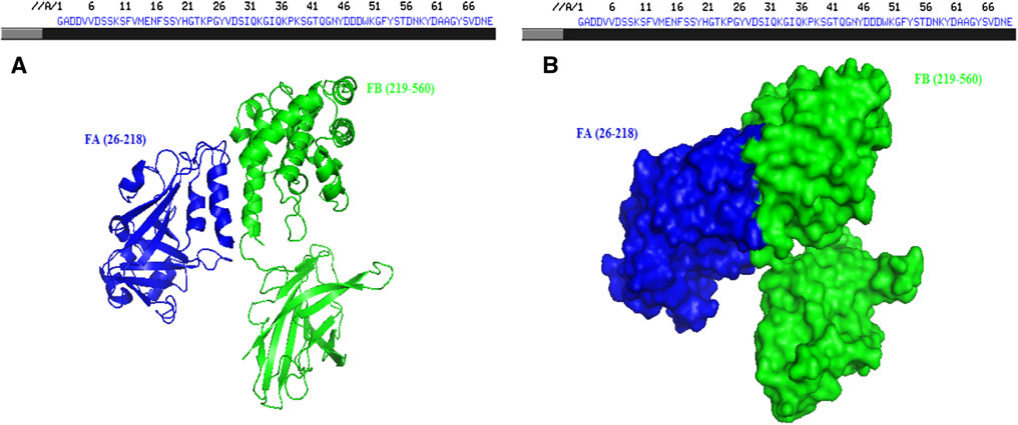
Folding (**a**) and surface (**b**) structures of FA and FB parts of diptheria toxin

The primary subunit of diptheria toxin was determined by *UniProtKB* database. By determining FA (26–218), FB (219–560) and signal peptide (1–25) which forms DT of the primary sequence, an PDB folder was created by academic version of MODELLER (18) which is homology modelling software. According to data in PDB folder, three dimensional folding structures of FA and FB fragments were displayed (Fig. 1).

#### Modelling of subunits of G-actin by using the primary sequences of It

The primary subunit of G-acitne which was obtained from rabbit skeleton muscle, was determined by *UniProtKB* database. Taken part in literature, it was determined that first two amino-acid mature form builds the main chain of actin between 3 and 375 sequences.

#### The primary sequence of actin obtained from rabbit muscle

**Fig. 2:**
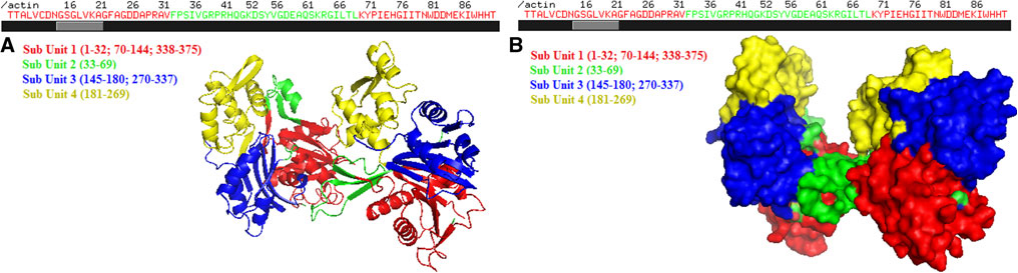
Folding (**a**) and surface (**b**) sturucture of G-actin

Formed by X-ray crystallography obtained from PDB protein bank (www.pdb.com), PDB folder of G-actin whose primary sequence was determined, was defined according to residues pyton algorythm, and folding structures were displayed (Fig. 2).

#### Estimation of possible interaction area between FA and G-actin

**Fig. 3:**
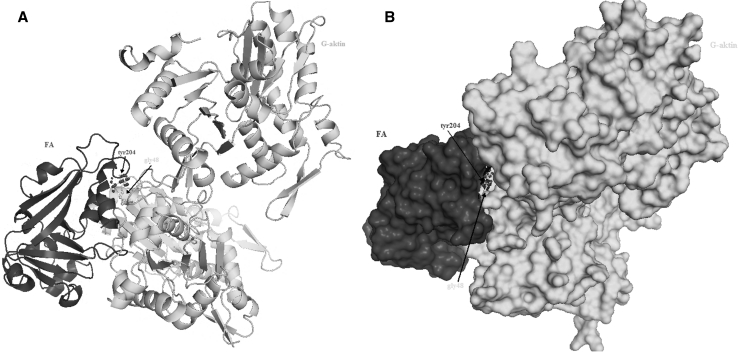
Folding (**a**) and surface (**b**) of ınteraction area between FA and G-actin

PDB folder data, belongs to FA part of G-actin and diptheria toxin whose structures and surfaces were determined before, was put into interaction at ClusPro 2.0 simulation software which is readily available on protein–protein docking system at Structural Bioinformatics Laboratories of Boston University. According to the results, most possible interaction PDB folder was created. Possible interaction area was displayed (Fig. 3).

According to thermodynamic calculations and sterechemical analysis of model, it was estimated that there might be a protein–protein interaction between tyr204 of FA and gly48 of G-actin. Surface analysis of the model was made and displayed (Fig. 3).

#### Increasing the purity of G-actin by chromotagrophic method

**Fig. 4:**
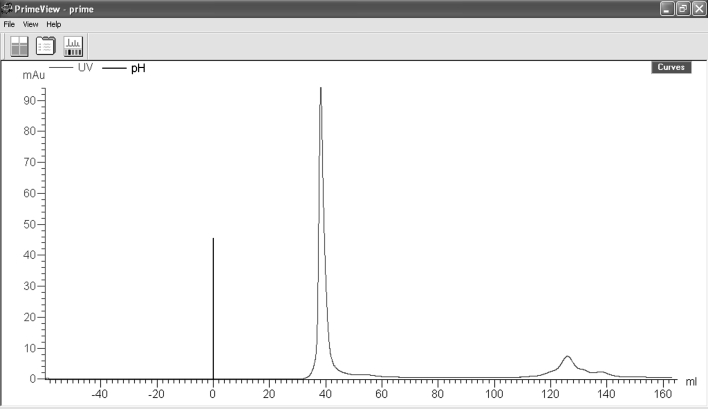
Chromotographic analysis of G-actin after centrifuge. After polymerization-depolymerization process, purity analysis of G-actin (0.5 ml or 2 mg) which was taken from supernatant liquid part (S120) after ultracentrifuge. Sample volume is 1 ml and flow rate is 0.8 ml/min

2 mM K-fosfat pH:7.5, 0.5 mM ATP, 0.1 mM CaCl_2_ 0.5 mM DTT, 1 mM NaN_3_, 50 mM Tris–HCl pH:7.4 was added to depolymerization buffer of F-actin which was obtained from rabbit striated muscle tissue, and lysed on glass homogenizer, then supernatant liquid (S-100) was resolved on ultracentrifuge by being centrifuged at 1000.000×*g* for 45 min. G-actin in supernatant liquid was purified by being put into chromotagrophy of gel filtration. The amount of it was spectrophotometrically measured via A_280_^0.1%^ = 1.25 formula [8]. Finally, the purity of G-actin was measured as 90 % (Fig. 4). Little rise at the back was thought to be resulted from ATP in buffer.

**Fig. 5:**
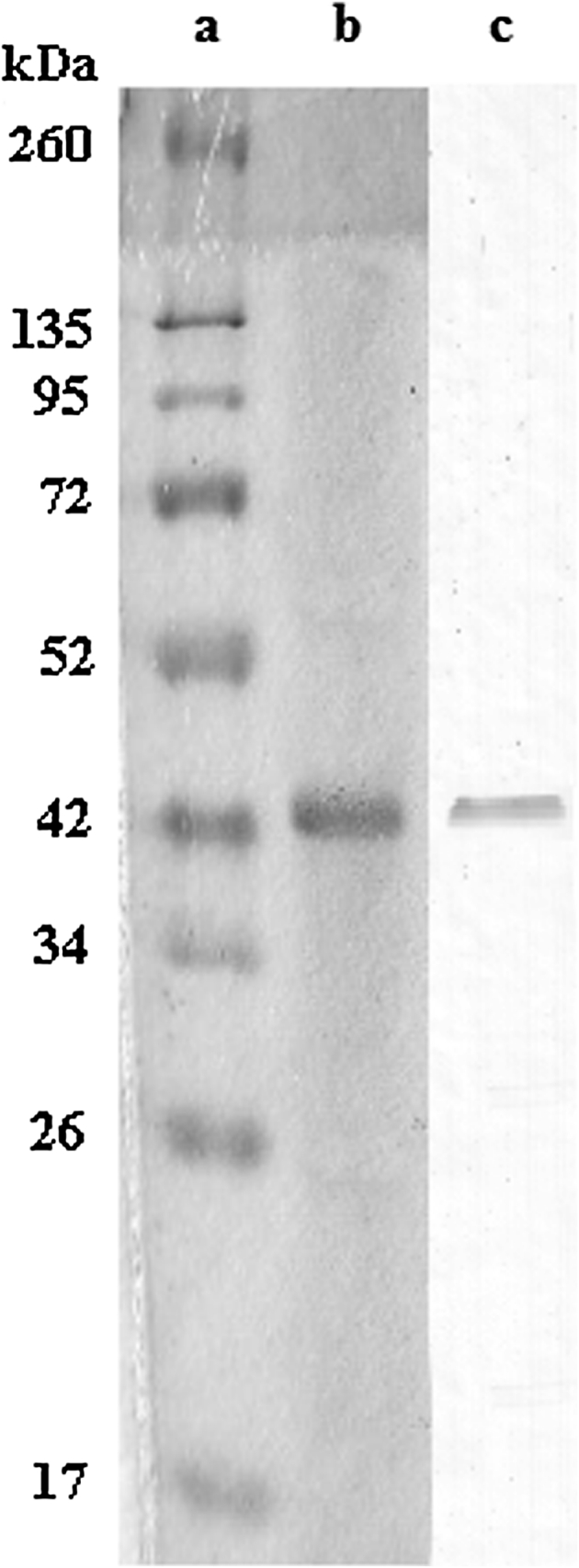
Display of G-actin by SDS-PAGE and ımmune blotting. **a** Molecular weight indicator (20 μg), **b** Purified G-actin after gel filtration (20 μg), **c** Display of actin of “b” after immune blotting in the presence of anti-actin

As mentioned in “Materials and methods” section, G-actin which was resolved from gel filtration chromatography system, was displayed by immune blotting analysis after SDS-PAGE gel electrophorese (Fig. 5b). In order to obtain chromogenic signal from nitrocellulose membrane where G-actin was moved, rabbit-improved anti-actin was used. Dilution rate of anticore was determined as 1:500. Membrane was waited with first anticore for 2 h. Dilution rate of second anticore (anti-rabbit IgG), goat-improved and alkaline phosphatase tagged, was determined as 1:1000 and applied to the membrane for 1 h. With addition of alkaline phosphatase substrate buffer, signal was seen on the membrane (Fig. 5c).

#### Purification of enzymatical part (FA) of diptheria toxin

**Fig. 6:**
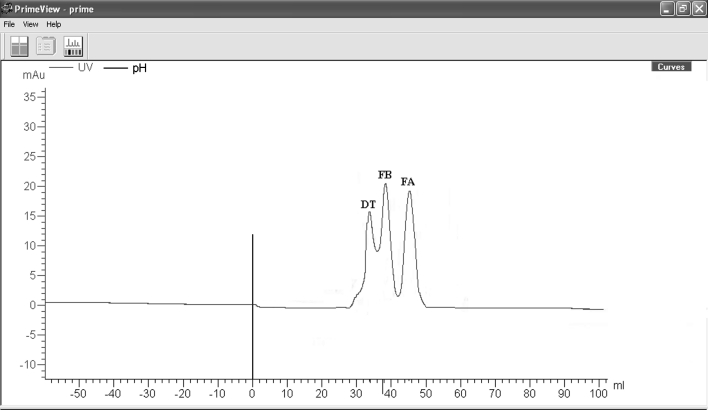
Segmentation of diphtheria toxin cut with tryspin in the system of gel filtration chromatography.After 500 μg toxin was toxined, it was put into the colon in 1 ml. Flow rate is 0.8 ml/min, sample volume is 1 ml

**Fig. 7:**
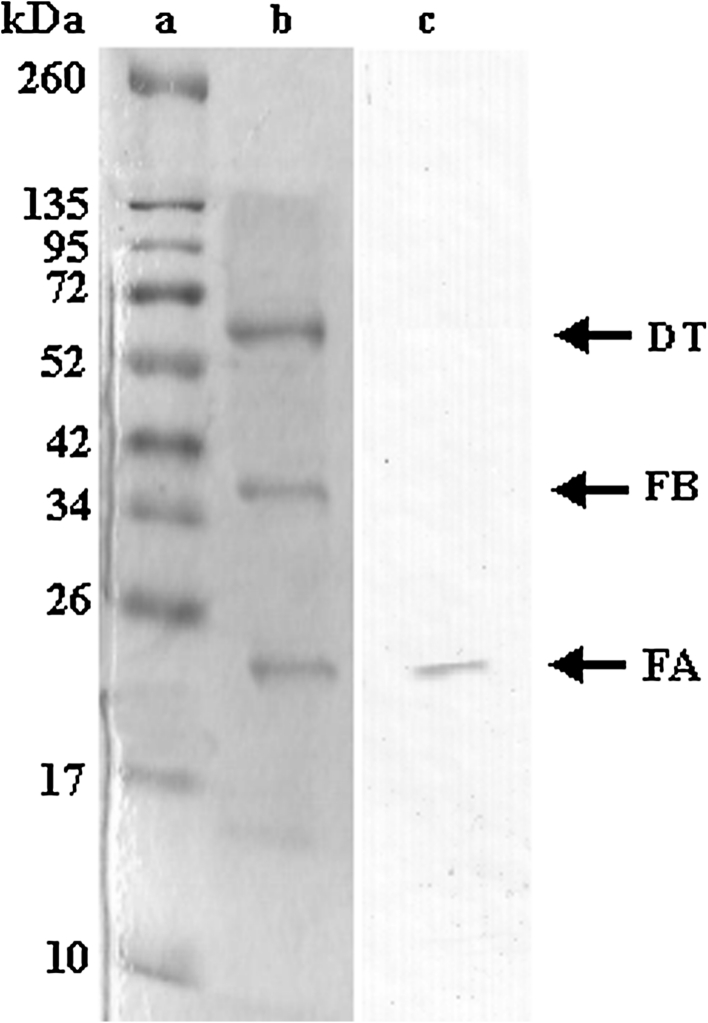
Display of diphtheria toxin with SDS-PAGE and immune blotting after partial digestion. **a** Molecular weight indicator (20 μg). **b** Tryspined diphtheria toxin (5 μg). **c** Immune blottting of “b” in the presence of anti-FA

As mentioned in “Materials and methods” section, diptheria toxin was saturated with tryspin at molar rate of 1/200 for 30 min. Trypthic pieces were resolved by chromatographic (Fig. 6) and electrophoretic methods (Fig. 7b). Moreover, FA was determined by even immune blotting method after electrophorese (Fig. 7c).

FA part of diptheria toxin, partially digested in the presence of tryspin which was an alternative to chromatographic method, was purified from gel after elecktrophorese. By adding 100 μl 6 M guanidin hydrochloride, it was dialysed during all night against renaturation solution (50 mM Tris–HCl pH 7.5, 150 mM KCl, 1 mM DTE, 0.1 mM EDTA, 0.1 mg/ml BSA, 20 % gliserol) and renaturized. It was concentrated in vivaspin tubes (V-10.000). Similar procedure was applied by purifying from salt after lyophilization and dialysis.

#### Determination of G-actin and FA interaction

**Fig. 8:**
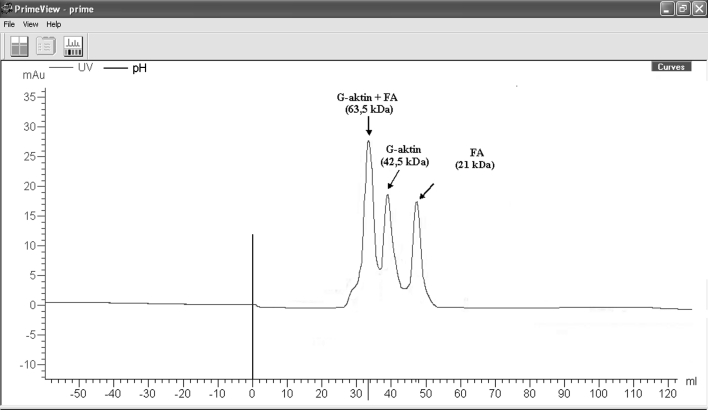
Interaction of G-actin-FA, flow rate is 0.8 ml/min, sample volume is 1 ml (G-actin 500 μl, FA 250 μl)

Purified from chromatography or gel, FA was put into interaction in bonding conditions and then mixture was resolved with gel filtration chromatography sephacryl S-100. After interaction of two proteins, it was observed that 63 kDa complexin was obtained. In addition, it was observed that both G-actin and FA peaks in their own area were decreased (Fig. 8).

**Fig. 9:**
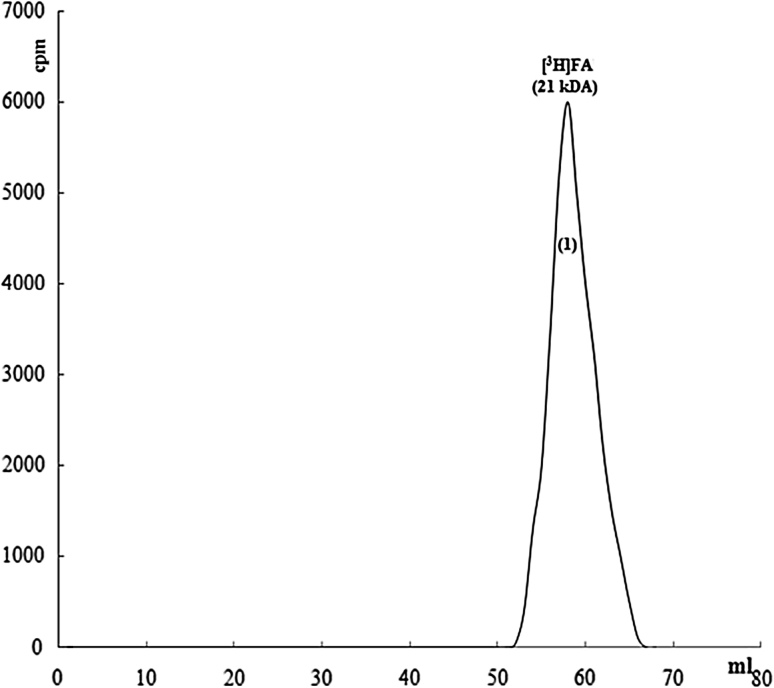
Chromatographic analysis of [^3^H]FA, flow rate is 0.8 ml/min, sample volume is 1 ml, loaded sample is 1 ml (FA 250 μl)

Similarly, it was tried that complex development, which would be interacted with G-actin and FA marked with ^3^H-sodium borohydryde, was displayed by chromatographic method. Therefore, 0.2 mg FA was marked in the presence of about 200 μCi [^3^H] sodium borohydryde at room temperature for 1 h. Free-radioactivity was removed after dialyse and radioactivity on FA was determined by calculating (Bray) in scintillation liquid. 20 μg/ml [^3^H]FA was put into gel filtration chromatography and radioactive content of sample was determined. In the region of about 21 kDa, highly purified and marked FA was resolved (Fig. 9).

**Fig. 10:**
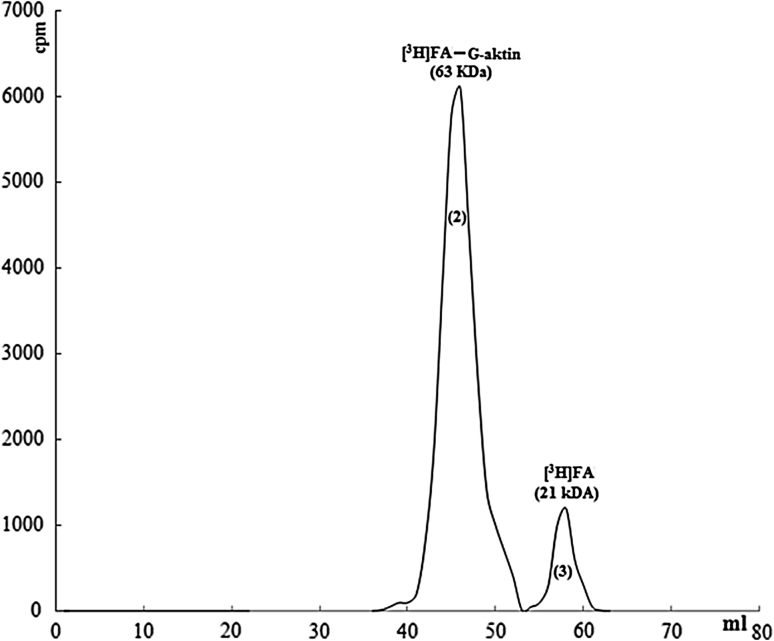
Chromatographic display of ınteraction of G-actin and radioactive-marked FA, flow rate is 0.8 ml/min, sample volume is 1 ml, loaded sample is 1 ml

In order to show the interation between G-actin and FA, radioactive-marked FA and G-actin were put into interaction in molar dimension at rate of 1/1 and in G-actin medium. Complex of G-actin-FA was seperated from gel filtration chromatography. Radioactive-marked FA was fallen in more anterior region in the colon (63 kDa). Marked FA was mainly determined as 90 % complexed (Fig. 10).

#### Removal of interaction of G-actin-FA

**Fig. 11:**
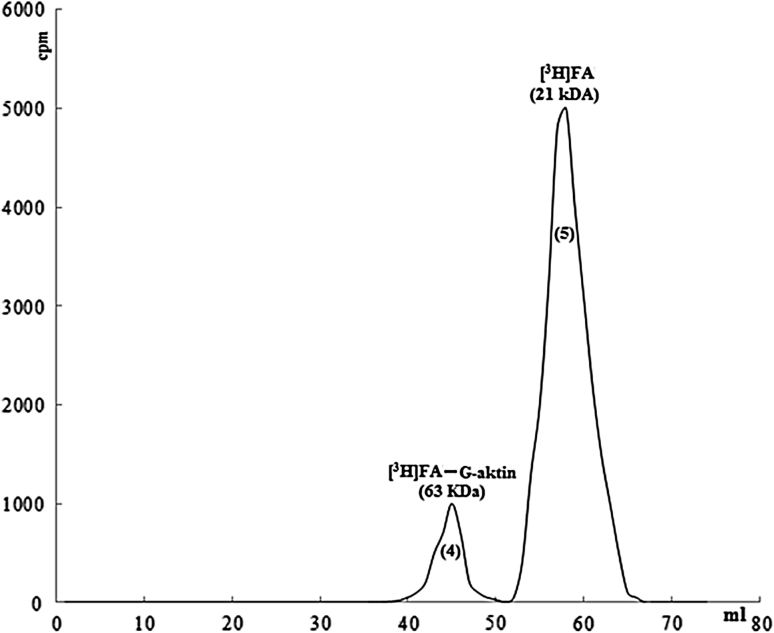
Removal of ınteraction of G-actin-FA in the presence of artificial peptide

**Fig. 12:**
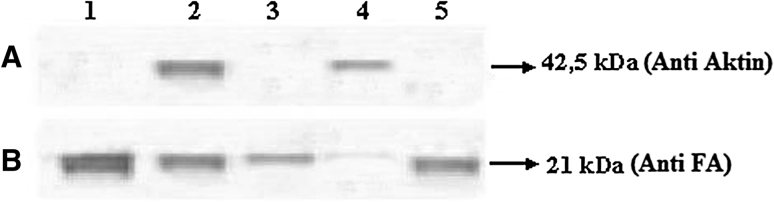
Interaction of G-actin-FA and ınhibition of the ınteraction. 2 μg protein was put into electrophorese. **a** Immune blotting in the presence of anti-actin, fraction 60 (*peak 1*) on Fig. 9, fraction 45 (*peak 2*) on Fig. 10, fraction 58 (*peak 3*) on Fig. 10, fraction 45 (*peak 4*) on Fig. 11, fraction 58 (*peak 5*) on Fig. 11, **b** immune blotting in the presence of antiFA, fraction 60 (*peak 1*) on Fig. 9, fraction 45 (*peak 2*) on Fig. 10, fraction 58 (*peak 3*) on Fig. 10, fraction 45 (*peak 4*) on Fig. 11, fraction 58 (*peak 5*) on Fig. 11

After computer simulation and thermodynamic calculations, possible interaction areas of G-actin and FA were calculated according to the lowest possibility from the highest possibility. Synthetic peptides which were imported, were appropriate for about 100 amino-acids area, were on second subunit on G-actin, and also each of them consisted 15 amino-acids. After, synthetic peptide (DAMYEYMAQACAGNR) (50 μg) which had highest bonding possibility among them, was put into interaction with G-actin (500 μg) at room tempretature for 2 h, and centrifuged in vivaspin tubes-10.000, peptides which were not bonded, were removed. In the next process, G-actin, on which peptide was bonded, was put into interaction with [^3^H]FA and removed from the colon. When radioctivity in samples was determined, it was observed that the complex (63 kDa), which was previously observed, was largely removed and radioactivity was on 21 kDa area (Fig. 11). Decrease in bonding was determined at rate of 80 or 90 % (Fig. 12).

Artificial peptide (250 μg), G-actin (500 μg), ^3^H marked FA (250 μg), flow rate is 0.8 ml/min, sample volume is 1 ml, loaded sample is 1 ml.

Chromatographic rises (1–5) on the purpose of interaction of [^3^H]FA-G-actin and removal of interaction in the presence of synthetic peptide, anti-actin after SDS-PAGE and immune blotting in the presence of anti-FA were displayed via immuno-blotting (Fig. 12).

As a result, the interaction of G-actin-FA which was theoretically determined in the region, was shown experimentally.

## Discussion

Diptheria toxin (DT) is highly effective and fatal with increased amount of DT. During the period following infection in heart muscle where actin, which is one of proteins forming cytoskeleton, intensively exists, and it has been observed that miotoxic effect has occured; and because of that, deaths resulting from heart attack have been reported [19]. It is thought that in the cells which are sensitive to DT, toxin stops protein synthesize because of toxin’s cytotoxic effect, and causes cell death by destructing cytoskeleton of the cell. DT interacting with the cell, is separated into two parts by reducing S–S bonds. Equal to N-terminus of DT, FA has an activity of ADP-ribozyltransferase. FB provides holotoxin to connect to the cell. After toxin is connected to the receptor on the surface of the cell (heparin-binding epidermal factor, HB-EGF), FA reaches to cytoplasm. Given off from endosome, FA causes to stop protein synthesis by ADP-ribosing eucaryotic elongation factor 2 (eEF2). Effect of toxin is not limited just with protein synthesis. It is known that the effect of toxin damages DNA fragmentation, apoptosis and cytoskeleton. It is thought that destruction of cytoskeleton occurs as a result of the interaction of FA with actin.

Previously, FA-actin interaction was displayed as in vitro and in vivo [20]. That FA broke the chain of F-actin was showed by using the techniques of chromatographic, electrophoretic and immunoflourescence. This study was planned to show the occuring of the interaction in which areas and through which amino-acids. First, the actin was purified from rabbit striated muscle tissue. FA,which has enzymatic activity, was purified from the gel occurring after SDS-PAGE and the gel filtration occurring in the process followed by cropping toxin in the presence of tryspin. Interaction of G-actin with FA in vitro conditions was displayed in gel filtration chromatography. A similar study was also carried out by radioactive-marking of FA. Experimental (chromatography, electrophorese and immune blotting) and theoretical (analogy modelling) methods were used in determining interaction area. Firstly, in order to close most possible areas where FA could connect with actin, synthetic peptides were used. 6 peptides which were special to FA’s connection areas, were interacted with G-actin, and so the bonding of FA was observed. As a result of our studies it was observed that 15 amino-acid artificial peptides (DAMYEYMAQACAGNR) which was equal to residual of 201–215 amino-acid of FA, interacted with secondary subunit of G-actin and closed this area. Secondly, in the model which was formed by analogy modelling, it was determined that most possible interaction would be between FA (tyr204) which was subunit of diptheria toxin, and G-actin (gly48) in the presence of synthetic peptides. It is known that Dnaz I known as one of actin-binding proteins, was bounded from amino-acids whose numbers were 41, 43 and 45 [21]. DNaz I which is one of the proteins organizing intracellular concentration of actin, interacts with actin at rate of one to one. Thus, nuclease activity stops. Interaction area of DNaz I-actin is equal to secondary subunit of actin (amino-acids between number 33 and 69) and shows a similar condition of actin-FA interaction, which we had as a result of our study. Secondary subunit of actin is equal to bearded edge of FA (+edge) and polymerization occurs at this edge ten times faster than the other edge. According to findings we obtained via experimental and theoretical methods, FA which connects to actin from bearded edge at secondary subunit, prevents polymerization of actin. These results bring to light mysterious features except for protein synthesis so far, and also partly explain biological relation in some measure. It has been long accepted that stopping protein synthesis is primary even only reason of ADP-ribosyltransferase activity [10] which is special to eEF2 of FA and cytotoxicity which FA causes [22, 23].

On the other hand, protein synthesis stopped because of FA’s channeling but there are cell kinds not resulting in cell deaths. Besides stopping protein synthesis, it was determined that cytochrome causes c’s releasing and caspase 3’s activation while DT causes destruction of cytoskeleton; internucleaseosmole causes lysis of DNA and cell lysis [23, 24].

Interaction of FA-actin whose biological importance was previously mentioned, should be illuminated within the frames of events following toxin’s enterance to the cell. New findings show that actin stops ADP-ribosylation of eEF2 channelled by FA [11]. This study explains that FA connected to actin can not start this reaction and possible intracellular accumulation of actin molecules found in high abundance can resist the effect of FA in the cell.

## Conclusions

It is known that moving diphteria toxin to intracellular is carried by endocythic process. Actin’s carrying support in toxin’s intracellular movement increases the importance of interaction of FA-actin. Both actin and eEF2 which is one of the actin-bonding proteins, support toxin’s releasing from endosomes besides the carrying of toxin. In this study, the interaction can be experimentally studied by cloning the areas both on FA (tyr204) and G-actin (gly48), and by changing with site-directed mutagenesis studies. In another approach, intracellular interactions of FA-cloned actin can be analysed with fluorescent techniques by using FRET technology.
